# Hydrogen Sulfide Delays LPS-Induced Preterm Birth in Mice via Anti-Inflammatory Pathways

**DOI:** 10.1371/journal.pone.0152838

**Published:** 2016-04-01

**Authors:** Weina Liu, Chen Xu, Xingji You, David M. Olson, Sylvain Chemtob, Lu Gao, Xin Ni

**Affiliations:** 1 Department of Physiology, Second Military Medical University, Shanghai, China; 2 Departments of Obstetrics and Gynecology, Pediatrics and Physiology, University of Alberta, Edmonton, Canada; 3 Departments of Pediatrics, Ophthalmology and Pharmacology, CHU Sainte-Justine Research Centre, Montréal, Canada; Fudan Univeristy School of Pharmacy, CHINA

## Abstract

A major cause of preterm labor in pregnant women is intra-amniotic infection, which is mediated by an inflammatory process. Hydrogen sulfide (H_2_S), a gaseous transmitter, has been implicated to be involved in inflammatory responses. We sought to investigate whether H_2_S affects infectious preterm birth using the mouse model of lipopolysaccharides (LPS)-induced preterm birth. Administration of LPS at 0.4 mg/kg with two injections intraperitoneally (i.p.) on gestational day 14.5 induced preterm labor. LPS significantly increased leukocyte infiltration in uterus, stimulated the expression of pro-inflammatory cytokines interleukin 1β (IL-1β), IL-6, tumor necrosis factor α (TNF-α), CCL2 and CXCL15 in myometrium. Administration of NaHS (i.p.) delayed the onset of labor induced by LPS in a dose-dependent manner. NaHS prevented leukocyte infiltration into intrauterine tissues and inhibited the production of pro-inflammatory cytokines in myometrium and decreased the levels of these cytokines in maternal circulation. H_2_S also decreased LPS-activated extracellular signal-regulated kinase (ERK) 1/2/ nuclear factor (NF)-κB signaling pathways in myometrium. This study provides new *in vivo* evidence for the roles of H_2_S in attenuating inflammation, and a potential novel therapeutic strategy for infection-related preterm labor.

## Introduction

Preterm birth (PTB) occurs in 5–15% of all pregnancies worldwide, and is the leading cause of infant morbidity and mortality [1, 2]. Of the survivors of PTB, 25% will have at least one developmental delay such as cerebral palsy and long term vision, hearing and respiratory problems [3]. Approximately half of PTB is of unknown aetiology, while maternal genito-urinary infections account for up to 30% to 40% of all PTB [4]. It has been demonstrated that uterine infection initiates a cascade of events such as induction of inflammatory responses, premature rupture of fetal membranes and myometrial contractions, all leading to preterm delivery of fetus [4].

There is increasing body of evidence indicating that the inflammatory state within uterus is a common element of both infection and idiopathic PTB [5, 6]. The expression of pro-inflammatory cytokines (including chemokines) in uterine tissues is increased prior to onset of term and preterm labor, and infiltration of myometrium, cervix, and fetal membranes by neutrophils and macrophages (Mϕ) [7–9] is found in both term and preterm labor. Many studies have demonstrated that inflammatory mediators such as IL-1β, IL-6 and TNF-α can stimulate the expression of contraction-associated proteins (CAPs), such as oxytocin receptor (OTR), connexin 43(CX43), prostaglandin H synthase (PGHS)-2 and prostaglandin receptors, in myometrium and production of uterotonic factors such as PGs, eventually leading to onset of labor. Thus, it has been implicated that these inflammatory pathways represent targets for the development of novel therapeutic agents to prevent PTB.

Hydrogen sulfide (H_2_S) has recently been suggested to be “the third endogenous gaseous signaling transmitter” in addition to nitric oxide (NO) and carbon monoxide (CO) in mammalian tissues. H_2_S has been implicated in many physiologic and pathologic processes, in particular, the inflammatory responses. There are many studies demonstrating that H_2_S exerts the anti-inflammatory actions in various tissues [10–12]. However, controversies emerge from many studies, indicating that H_2_S may play dual roles in the inflammatory process. For instance, H_2_S in synovial fluid appears to act as a pro-inflammatory mediator in rheumatic diseases [13]. In the U937 human monocyte cell line, NaHS causes enhanced TNF-αand IL-1β output via activation of extracellular signal-regulated kinase 1/2 (ERK1/2)/nuclear factor-κB (NF-κB) signaling pathway [14]. It has been suggested that the concentration/production rate of H_2_S, physiological/pathological condition and cell/tissue type contribute to divergent effects of H_2_S on the inflammatory responses [15–18].

A number of studies have demonstrated that gestational tissues, including placenta, fetal membranes and myometrium, express H_2_S generating enzymes cystathionine-β-synthase (CBS) and cystathionine-γ-lyase (CSE) [19–21]. Our previous study has shown that H_2_S suppresses contractions in human myometrial strips *in vitro* [22], suggesting that H_2_S might be involved in labor process. More recently, it has been implicated that gasotransmitters might be a solution for the therapeutic dilemma for pregnancy diseases such as preeclampsia [23]. Thus, it would be of interest to explore the effect of H_2_S on PTB. As mentioned, inflammatory processes play significant roles in most, if not all, labors regardless of the presence of infection, and H_2_S is involved in regulation of inflammation in various tissues. We investigated whether H_2_S affects the timing of birth in a LPS-induced preterm labor mouse model and examined inflammatory profile in gestational tissues of the mice.

## Materials and Methods

### Animals

Mice of the BALB/c strain (20–25 g body weight, 6–8 weeks old) were obtained from Shanghai SLAC Laboratory Animal Co (Shanghai, China). They were housed in groups in the cages under controlled conditions of light (12 h light, 12h dark) and temperature (23–25°C). Animals received pellet chow and water ad libitum. All animal experiments were approved by the Ethical Committee of Experimental Animals of Second Military Medical University, and in accordance with the National Institutes of Health Guide for the Care and Use of Laboratory Animals. The animals were observed at least once daily before they were mated. The criteria we used to monitor animal health include the condition of coat and skin, the appearance of the eyes, the gait pattern, self-biting, self-clasping, self-grasping, hair pulling-and eating,face or eye poking et al.

**Fig 1:**
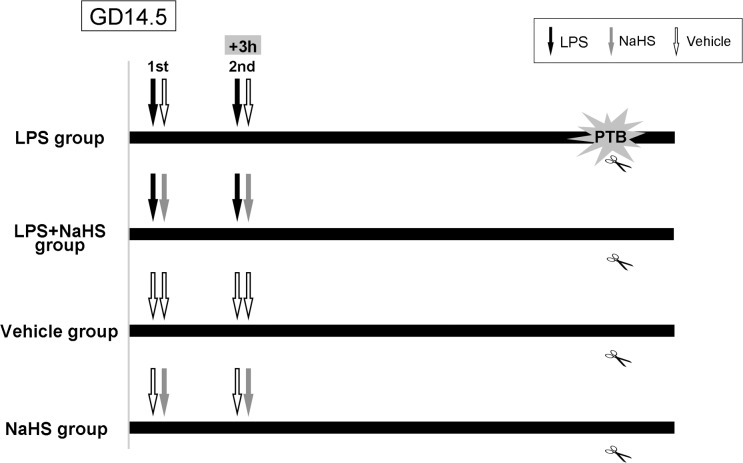
Diagram for the animal model procedure. Timed-pregnant mice at 14.5 dpc were injected intraperitoneally (i.p.) with LPS with or without NaHS at different doses in 100μl NS twice a day at 8:30 and 11:30. Labor time was considered as the first pup was delivered. GD: gestational day. Black arrow: LPS i.p. Grey arrow: NaHS i.p. White arrow: NS i.p.

Female mice were housed with male mice overnight beginning at 18:00. Mice found to have vaginal plugs at 6:00 the following day were considered to be 0.5 day post-coitum (dpc). Timed-pregnant mice at 14.5 dpc were injected i.p. with *Escherichia coli* 0111:B4 lipopolysaccharides (LPS, Sigma-Aldrich, St. Louis) at 0.2, 0.4 or 0.8mg/kg in 100μl sterile normal saline (NS) with or without sodium bisulfide (NaHS, Sigma-Aldrich) at 5, 7.5, 10, 15mg/kg in 100μl NS twice a day at 8:30 and 11:30 (Fig 1). Control pregnant mice at 14.5 dpc received 100μl NS with or without NaHS at 10mg/kg twice a day at 8:30 and 11:30. They were monitored every 2 hours after first LPS-injection to see if they were in labor. Besides signs mentioned above, the size of belly, the blood or remnant of placenta and neonatal mice were also monitored for pregnant mice. No animals from the control group became severely ill or died prior to the experimental endpoint. However, two from six animals injected with 15mg/kg NaHS were euthanized via cervical dislocation, due to apparent vaginal bleeding and threatened abortion prior to active labor.

Myometrium, placenta and blood samples from the mice receiving LPS injection were collected at 1h, 2h, 4h, 5h post-injection and the time when LPS-treatment group delivered first pup (~8h after LPS injection) within 5 min under anesthesia with 10% chloral hydrate (5μl/g, i.p.). In this case, no side effect of anesthesia to fetus and mother was observed. Samples of control pregnant mice were collected at 8h post-injection. Myometrium was isolated after removal of all fetal-derived tissues, and the endometrial layer was also removed by gently scraping and blotting. The myometrium and placenta were then rinsed in ice-cold 1× PBS, flash frozen in liquid nitrogen and stored at -80°C along with the maternal serum until analysis.

### Enzyme-linked Immunosorbent Assay (ELISA)

The concentrations of IL-1β, IL-6, TNF-α, CCL2 and CXCL15 in mouse uterine tissue and maternal serum were determined by specific enzyme-linked immunoassay kit (Westang Biotech Co Ltd, Shanghai, China), according to the manufacturer’s instructions.

### Immunohistochemistry

Mouse placenta and surrounding uterus were fixed in 4% paraformaldehyde solution and embedded in paraffin. Paraffin sections (5 μm) were cut, rehydrated and microwaved in citric acid buffer to retrieve antigens. Then, the sections were treated with 3% hydrogen peroxide (H_2_O_2_), followed by 10% normal goat serum blocking at room temperature for 30 min, then incubated with CD45 antibody (Santa-Cruz, Dallas, TX; 1:200) diluted in PBS containing 1% BSA for 24h at 4°C. The sections were incubated with mouse anti rabbit IgG for 30min at room temperature after washing with PBS (3min×3 times). The signals were detected with the biotin–streptavidin–peroxidase system using diaminobenzidine (Sigma-Aldrich) as chromogen. Negative controls were performed with primary antibody replaced by PBS.

### Quantitative real-time RT-PCR

Total RNA of myometrial tissues was extracted by TRIzol reagent (Invitrogen Corp., Carlsbad, CA) following the manufacturer’s instructions. The RNA samples were treated with DNase (Invitrogen) to remove any contaminating DNA. 2 μg RNA was reversed transcribed using random primers and Moloney Murine Leukemia Virus Reverse Transcriptase (MMLV, Promega, Madison, USA). All PCR primers were synthesized by Sangon Biotech (Shanghai, China) and the sequences of the primers were shown in S1 Table. Quantitative real-time PCR analysis was carried out in duplicates using CFX Connect Real-Time PCR Detection System (Bio-Rad, Singapore). Real-time PCR solution consisted of 40 ng diluted cDNA product, 0.1–0.3 μM of each paired primer and SYBR Green PCR Master Mix (Aidlab, Shanghai, China). PCR conditions were optimized according to preliminary experiments to achieve linear relationships between initial RNA concentration and PCR product. The specificity of the primers was verified by examining the melting curve and efficiency was validated by standard curve. The amplification cycles were set at 40 cycles. Amplification of the housekeeping genes β-actin and GAPDH were measured for each sample as an internal control for sample loading and normalization. To determine the relative quantitation of gene expression for both target and housekeeping genes, the comparative Ct (threshold cycle) method with arithmetic formulae (2^-ΔΔCt^) was used [24]. Because very similar data were obtained by using either β-actin or GAPDH as an internal control, GAPDH was used for calculation of ΔCt in presentation of results.

### Western Blotting

Approximately 50mg of myometrial tissues were homogenized in cold RIPA lysis buffer containing protease/phosphatase inhibitor cocktail (Roche, Mannheim, Germany) and PMSF. Tissue homogenates were sonicated in an ice bath and then boiled at 100°C for 5 min. Protein samples (30μg) were separated by 10% SDS-PAGE gel electrophoresis and subsequently transferred to nitrocellulose membranes. After blockage in 5% skim milk, membranes were incubated with specific primary antibodies including phospho-p65 (CST, Boston, MA), p65 (CST), phospho-ERK1/2 (CST) and ERK1/2 (CST) under constant shaking overnight at 4°C. The membranes were then incubated with a secondary horseradish peroxidase-conjugated antibody for 1 h at room temperature. Immunoreactive proteins were visualized using the Enhanced Chemiluminescence Western blotting detection system (Millipore, Billerica, USA). The chemiluminescent signals from the membranes were quantified by a Gene Gnome HR scanner (Synoptics Ltd, UK). The levels of phospho-p65 and phospho-ERK1/2 were normalized to the unphosphorylated form of these proteins, while the levels of unphosphorylated form of ERK1/2 and p65 were normalized to β-actin.

### Statistics

All the results are presented as mean±SEM. All the data were tested for homogeneity of variance by Bartlett’s test before analyzing the significance. Data were then analyzed by one-way ANOVA followed by Student-Newman-Keuls (SNK) multiple comparison method. In all of the tests, *P* <0.05 was considered to be significant.

## Results

### H_2_S delays onset of LPS-induced preterm labor

The animals received two injections of NS delivered by 124.38±6.76 hrs after the first injection (Table 1), which is comparable to the normal delivery time of mice. LPS administration dose-dependently reduced the time to delivery, with mice treated with both 0.4mg/kg and 0.8mg/kg delivering significantly earlier compared with the NS control group (0.4mg/kg LPS mean time to delivery: 7.67±0.89 h, p<0.001 compared with NS control group; 0.8mg/kg LPS mean time to delivery: 6.75±0.67 h, p<0.001 compared with NS control group; Table 1). Subsequent experiments were performed with 0.4mg/kg LPS, as this dose resulted in reliable preterm delivery with the least variation in time to delivery between mice and the minimal maternal and fetal mortality.

**Table 1:** Injection of LPS induced preterm labor of mice.

Treatment	N	Injection-to-delivery Interval (hours)
Normal Saline	12	124.38±6.76
LPS 0.2mg/kg	12	95.67±49.21
LPS 0.4mg/kg	12	7.66±0.89 ^a^
LPS 0.8mg/kg	12	6.75±0.67 ^b^

a: p = 2.01×10^−6^ compared with NS group

b: p = 1.9×10^−6^ compared with NS group

Various doses of NaHS (5mg/kg-15mg/kg) were then simultaneously injected with LPS at 2 time points, and the delivery time was monitored. Notably, administration of NaHS (10 mg/kg, i.p.) alone at 14.5 dpc with two injections did not change the timing of spontaneous labor in pregnant mice (S2 Table). As shown in Table 2, administration of NaHS at the dosage of 5mg/kg/injection with LPS did not affect the timing of birth and the incidence of PTB, with the ‘injection-to-delivery interval’ of 7.58±0.53 hrs. Administration of NaHS at the dosage of 7.5mg/kg/each injection did not influence the incidence of LPS-induced PTB, but delayed the onset of LPS-induced preterm labor from 7.58±0.53 hrs to 9.21±1.26 hrs (P = 0.030). NaHS at the dosage of 10 mg/kg/injection significantly delayed the onset of preterm labor from 7.58±0.53 hrs to 16.75±5.95 hrs (P<0.001). NaHS at the dosage of 15 mg/kg/injection could increase the maternal mortality (from 0 to 33.3%), so NaHS was used at 10 mg/kg in the subsequent experiments

**Table 2:** NaHS delayed the onset of LPS-induced preterm labor.

Treatment(mg/kg)	N	Injection-to-delivery Interval (hours)
LPS 0.4 + NS	12	7.67±0.89
LPS 0.4 + NaHS 5	12	7.58±0.53 ^a^
LPS 0.4 + NaHS 7.5	12	9.21±1.26 ^b^
LPS 0.4 + NaHS 10	12	16.75±5.95 ^c^
LPS 0.4 + NaHS 15	6	7.88±0.41 ^d^^,^ ^&^

a: p = 0.749 compared with LPS + NS group

b: p = 0.030 compared with LPS + NS group

c: p = 0.000 compared with LPS + NS group

d: p = 0.681 compared with LPS + NS group

&: After injection, two pregnant mice died before active labor.

### H_2_S attenuates LPS-induced leukocyte infiltration in decidua

**Fig 2:**
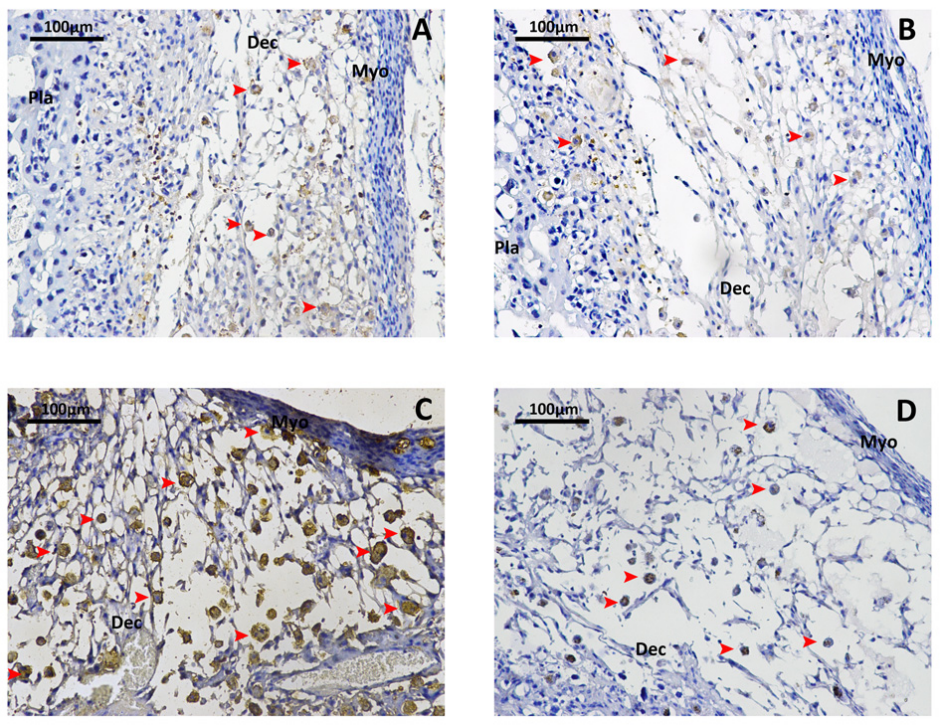
NaHS reverses the LPS-induced leukocytes infiltration into decidua. Total density of leukocytes in mice maternal-fetus unit was determined by immunostaining for CD45 (common leukocyte antigen). A. Mice injected with vehicle at 14.5 dpc. B. Mice injected with NaHS(10mg/kg) at 14.5 dpc. C. Mice injected with LPS (0.4mg/kg) at 14.5 dpc. D. Mice injected with LPS (0.4mg/kg) and NaHS (10mg/kg) at 14.5 dpc. Arrow heads indicate positively stained leucocytes. Dec, decidua; Pla, placenta; Myo, myometrium.

As shown in Fig 2, LPS dramatically enhanced the leukocyte infiltration in decidua, evident by the increased density of CD45+ cells compared to the mice received NS injection. Administration of NaHS (10mg/kg/each injection) significantly reversed LPS-induced leukocyte infiltration in decidua.

### H_2_S decreases maternal circulatory level of cytokines and chemokines in LPS-treated mice

**Fig 3:**
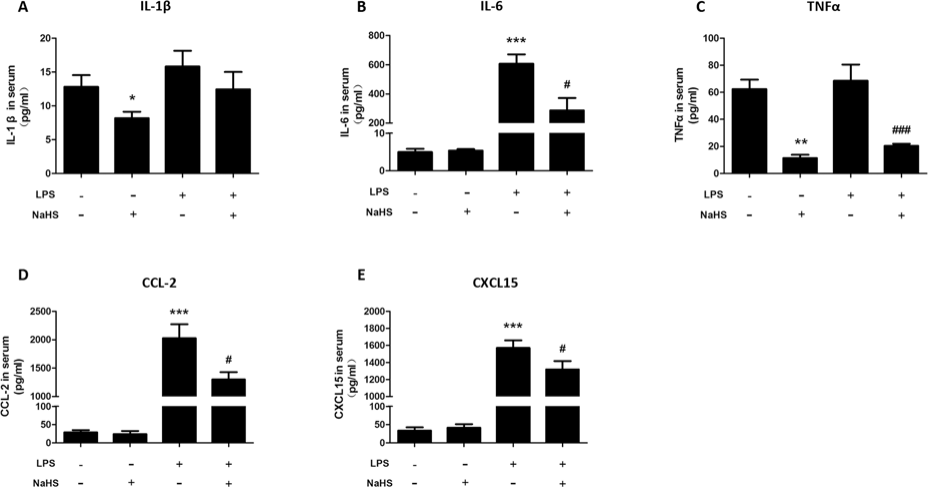
NaHS decreases maternal circulatory levels of cytokines and chemokines in LPS-induced preterm labor mice. The blood was harvested when the LPS-injected mice delivered the first pup. Serum was collected after centrifuge. ELISA was employed to determine the concentration of IL-1β (A), IL-6 (B), TNF-α (C), CCL-2 (D) and CXCL-15 (E). Data are presented as mean ± SEM. N = 6 (vehicle and NaHS groups) or 10 (LPS and LPS+NaHS groups).*P<0.05, **P<0.01, ***P<0.001 compared with vehicle control group, #*P*<0.05, ### *P*<0.001 compared with LPS-injected group.

As shown in Fig 3, LPS treatment dramatically increased the level of IL-6, CCL-2 and CXCL-15 (mouse homologue of IL-8) in maternal circulation at the time of onset of labor. Administration of NaHS (10mg/kg/injection) could significantly decrease maternal levels of IL-6, TNF-α, CCL-2 and CXCL-15 in mice received LPS treatment. In addition, administration of NaHS (10mg/kg/injection) also decreased maternal levels of IL-1β and TNF-α in control mice.

### H_2_S decreases mRNA expression of cytokines and chemokines in myometrium and placenta of LPS-treated mice

**Fig 4:**
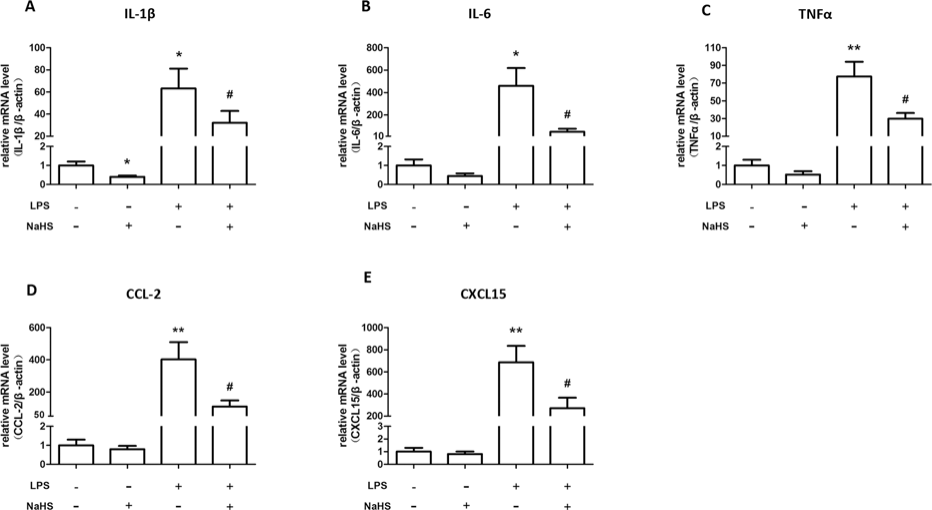
The effect of NaHS on the mRNA expression of cytokines and chemokines in myometrium of the normal pregnant and LPS-induced preterm labor mice. Mice accepted two i.p. injections of NS or LPS (0.4mg/kg) with or without NaHS (10mg/kg) at 14.5 dpc. The myometrium was harvested when the LPS-injected mice delivered the first pups. The mRNA levels of IL-1β (A), IL-6 (B), TNF-α (C), CCL-2 (D), and CXCL-15 (E) were measured by real-time RT-PCR and normalized by β-actin. Values are presented as mean ± SEM. N = 6 (vehicle and NaHS groups) or 10 (LPS and LPS+NaHS groups). * P<0.05, **P<0.01 compared with vehicle control, #: P<0.05 compared with LPS-injected group.

**Fig 5:**
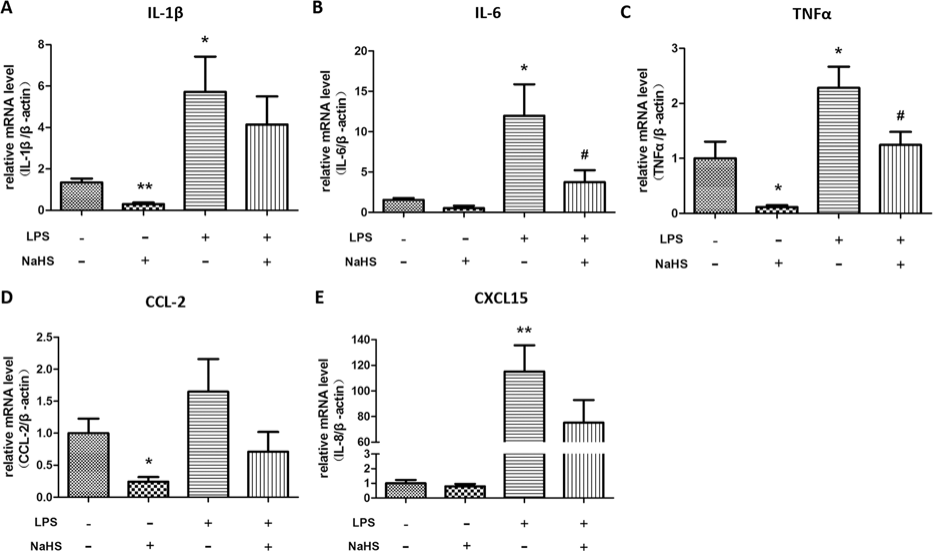
The effect of NaHS on the mRNA expression of cytokines and chemokines in placenta of the normal pregnant and LPS-induced preterm labor mice. The placenta was harvested when the LPS-injected mice delivered the first pups. The mRNA levels of IL-1β (A), IL-6 (B), TNF-α (C), CCL-2 (D), and CXCL-15 (E) were measured by real-time RT-PCR and normalized by β-actin. Values are presented as mean ± SEM. N = 6 (vehicle and NaHS groups) or 10 (LPS and LPS+NaHS groups). * P<0.05, **P<0.01 compared with vehicle control, #: P<0.05 compared with LPS-injected group.

Moreover, LPS treatment dramatically increased the mRNA levels of IL-1β, IL-6, TNF-α, CCL-2 and CXCL-15 in myometrium, which can be partially reversed by the injections of NaHS (10mg/kg, twice) (Fig 4). In placenta, LPS induced much milder yet significant increases in mRNA levels of IL-1β, IL-6, TNF-α, CCL-2 and CXCL-15, which also can be reversed by NaHS injection (Fig 5).

### H_2_S attenuates LPS-induced activation of ERK1/2 and NF-κB signaling pathway in myometrium

**Fig 6:**
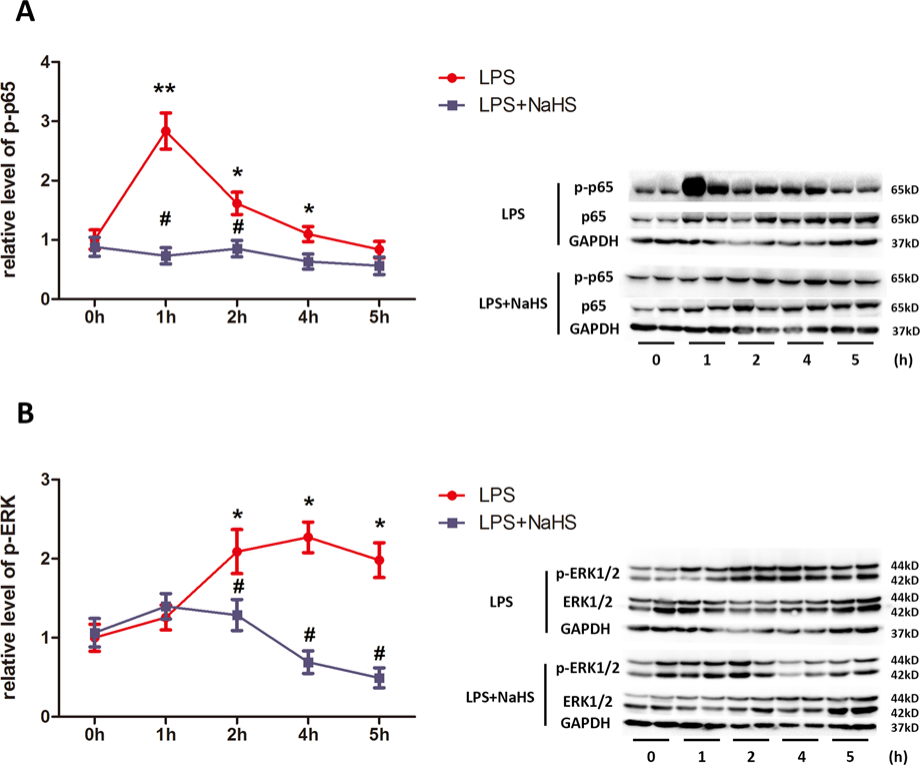
NaHS attenuates LPS-induced activation of ERK1/2 and p65 in myometrium. The uterine tissues were harvested 1h, 2h, 4h and 5h after LPS and/or NaHS injection. The levels of phosphorylated p65, p65, phosphorylated ERK1/2 and ERK1/2 were determined by western blotting. Phosphorylated p65 (A) and ERK1/2 (B) were normalized by p65 and ERK1/2. Values are presented as mean ± SEM. N = 4. * P<0.05, **P<0.01 compared with vehicle control group, #P<0.05, ##P<0.01 compared with LPS-injected group.

It is known that LPS activates various signaling pathways, such as ERK1/2 and NF-κB [25]. We therefore examined dynamic changes of these signaling pathways at 1h, 2h, 4h, and 5h after the first injection of LPS. As shown in Fig 6A, phosphorylated p65 was dramatically increased by LPS at 1h, remained elevated until 4h after LPS injection, and then returned to basal levels at 5h. Phosphorylated ERK1/2 was increased by LPS at 2 h and remained elevated until 5h after injection (Fig 6B). NaHS (10mg/kg/injection) significantly reversed LPS activation of p65 and ERK1/2 at all the time points observed (Fig 6A & 6B, blue lines).

## Discussion

In the present study, we demonstrated that H_2_S significantly delayed LPS-induced preterm labor and suppressed LPS-induced inflammation in myometrium. These data indicate that H_2_S is a potential therapeutic agent for infectious preterm labor.

Many studies have demonstrated that systemic administration of LPS induces preterm birth in rodents [26, 27]. Shynlova et al [27] recently shown that LPS (i.p.) administration on 14.5 dpc could significantly increase mRNA levels of pro-inflammatory cytokines in uterus and induce leukocyte infiltration in uterus in pregnant mice. In consistent with the above studies, we also demonstrated that administration of LPS on 14.5 dpc could induce preterm labor, leukocyte infiltration, mRNA expression of pro-inflammatory cytokines including IL-1β, IL-6, TNF-α, CCL-2 and CXCL-15 in myometrium and increased IL-6, CCL2 and CXCL-15 protein levels in maternal circulation. It is known that LPS binds to toll-like receptor 4 (TLR4) and subsequently activates multiple signaling pathways such as NF-κB and ERK 1/2 [25]. Consistently, we found that LPS treatment increased the levels of phosphorylated p65 and ERK1/2 in myometrium.

Although two recent studies demonstrated that neutrophil depletion fails to affect inflammation-induced preterm birth [28, 29], the role of other immune cell populations known to influx in association with parturition remains unclear. Previous studies have reported that the depletion of uterine natural killer (NK) cells[30] or macrophages[31] led to significant reductions in LPS-induced preterm birth, CD45 is a wide spectrum marker for leukocytes, which was shown to significantly enrich in decidua in LPS injected mice, and was reversed by H_2_S treatment. It is likely that different inflammatory stimuli will induce a different immune response. Future study using more specific markers to differentiate macrophages and NK cells infiltration under this circumstance will be needed to specify the immune cell population contributing to labor affected by H_2_S. As mentioned, the role of H_2_S in inflammation is controversial, with both pro- and anti-inflammatory effects described [11, 17, 32]. The present study showed that NaHS suppressed LPS-induced expression of pro-inflammatory cytokines in myometrium, decreased the level of pro-inflammatory cytokines in circulation and inhibited leukocyte infiltration and activation of NF-κB and ERK 1/2 signaling pathways in myometrium. These data strongly suggest that H_2_S delays LPS-induced preterm labor via inhibition of inflammation.

It should be pointed out that the inhibitory effect of NaHS on LPS-induced preterm birth was dose-dependent within the dosages of 5-10mg/kg. Higher dose (15mg/kg) of NaHS had no effect on gestational length in LPS-treated mice, instead, increased the maternal mortality. Actually, a number of studies have demonstrated that the effects of H_2_S on inflammation are dependent on the concentration of H_2_S [15–18]. NaHS releases H_2_S instantaneously in aqueous solution at striking dosage, but won’t maintain its concentration during extended period, which may account for its incapacity to completely reverse the LPS-induced preterm birth. Collectively, the relative low margin of safety and intrinsic characteristic of NaHS will largely limit its clinical use as an ideal H_2_S donor. The emerging new H_2_S donors (e.g. GYY4137)[33] with wider margin of safety and better mimicking the biological effects of naturally produced H_2_S *in vivo* need to be tested in future studies, and might become the more promising treatment for LPS-induced preterm labor.

Our previous study has shown that H_2_S suppresses spontaneous contractions in human myometrial strips [22], suggesting that H_2_S may play a role in the timing of the initiation of labor in human. However, in the present study, it was found that administration of NaHS (10mg/kg) with two injections did not affect the gestational length in pregnant mice. However, it was found that NaHS (10mg/kg) could decrease circulatory level of IL-1β and TNF-α and mRNA expression of IL-1β and IL-6 in myometrium. Since higher dose of NaHS can lead to maternal mortality, we did not examine the effect of higher dose of NaHS on gestational length in pregnant mice. Whether H_2_S plays physiological roles in the initiation of labor in mice needs to be further investigated.

Although H_2_S is an essential mediator of many biological functions, the underlying molecular mechanisms of its actions are still poorly understood. S-sulfhydration (SSH) of cysteine residues in proteins has now been recognized as one of the key mechanisms mediating physiologic roles of H_2_S [34–36]. Sen et al [37] found that H_2_S sulfhydrates the p65 subunit of NF-κB at cysteine-38, which augments its ability to bind the coactivator ribosomal protein S3 and stimulate transcription of anti-apoptotic genes. The SSH at the same cysteine residue or other sites of p65 may elicit the transcriptional activation of pro-inflammatory genes, which clearly awaits further investigation. Moreover, previous study showed that the modified cysteine in p65 becomes a target for S-nitrosylation, which exerts the opposite effects on anti-apoptotic genes [37]. It will be of interest to determine whether the reciprocal interaction between S-sulfhydration and S-nitrosylation also exists in the regulation of pro-inflammatory genes in myometrium.

In conclusion, the present study indicates that H_2_S significantly delays preterm labor induced by LPS through the inhibition of inflammation in an ERK1/2-NF-κB dependent manner. The study reveals new *in vivo* evidence for the roles of H_2_S in attenuating inflammation, and provides a potential novel therapeutic strategy for infection-related preterm labor.

## Supporting Information

S1 Table Primer sequences used for Realtime PCR. (DOCX)

S2 Table Injection of NaHS alone didn’t affect the onset of labor. (DOCX)
