# Supplementary material for: Hydrogen Sulfide Delays LPS-Induced Preterm Birth in Mice via Anti-Inflammatory Pathways
Source: PLoS One. 2016 Apr 1;11(4):e0152838. doi: 10.1371/journal.pone.0152838 (PMC4817991; doi:10.1371/journal.pone.0152838)
Supplement: S1 Table — (DOCX) [file pone.0152838.s001.docx]

**S1 Table. Primer sequences used for Realtime PCR**

| **Target genes** | **Primer sequences** | **GenBank accession No.** |
| --- | --- | --- |
| *IL-1β* | 5’- tgccaccttttgacagtgatg-3’  5’-aaggtccacgggaaagacac-3’ | NM_008361.3 |
| *IL-6* | 5’-cggccttccctacttcacaa-3’  5’-ttctgcaagtgcatcatcgt-3’ | NM_031168.1 |
| *TNFα* | 5’-aggcactcccccaaaagatg-3’  5’- gctcctccacttggtggttt-3’ | NM_001278601.1 |
| *CCL-2* | 5’-caccagccaactctcactgaa-3’  5’- cattccttcttggggtcagc-3’ | NM_011333.3 |
| *CXCL15* | 5’-ccgtccctgtgacactcaag-3’  5’-acagaagcttcattgccggt-3’ | NM_011339.2 |
| *β-actin* | 5’-ctgtatgcctctggtcgtac-3’  5’-tgatgtcacgcacgatttcc-3’ | NM_007393.3 |
| *GAPDH* | 5’-tctctgctcctccctgttct-3’  5’- gatggtgatgggtttcccgt -3 | NM_001289726.1 |
