# Supplementary material for: Hydrogen Sulfide Delays LPS-Induced Preterm Birth in Mice via Anti-Inflammatory Pathways
Source: PLoS One. 2016 Apr 1;11(4):e0152838. doi: 10.1371/journal.pone.0152838 (PMC4817991; doi:10.1371/journal.pone.0152838)
Supplement: S2 Table — (DOCX) [file pone.0152838.s002.docx]

**S2 Table. Injection of NaHS alone didn’t affect the onset of labor**

| **Treatment** | **N** | **Injection-to-delivery Interval (hours)** |
| --- | --- | --- |
| Normal saline | 12 | 124.38±6.76 |
| NaHS 5mg/kg | 12 | 123.83±6.84^a^ |
| NaHS 7.5mg/kg | 12 | 122.67±8.05^b^ |
| NaHS 10mg/kg | 12 | 122.5±7.11^c^ |
| NaHS 15mg/kg | 12^&^ | 124.67±6.80^d^ |

a: p=0.860

b: p=0.578

c: p=0.542

d: p=0.930

&:Three mice died after injection
